# Crack Shape Coefficient: Comparison between Different DFOS Tools Embedded for Crack Monitoring in Concrete

**DOI:** 10.3390/s23020566

**Published:** 2023-01-04

**Authors:** Tomasz Howiacki, Rafał Sieńko, Łukasz Bednarski, Katarzyna Zuziak

**Affiliations:** 1 Faculty of Civil Engineering, Cracow University of Technology, Warszawska 24, 31-155 Kraków, Poland; 2 Faculty of Mechanical Engineering and Robotics, AGH University of Science and Technology in Kraków, Mickiewicza 30, 30-059 Krakow, Poland; 3 Nerve-Sensors, Libertów, ul. Jana Pawła II 82A, 30-444 Kraków, Poland

**Keywords:** distributed fibre optic sensing, composite sensors, monolithic sensors, layered cables, sensing cables, strains, cracks, crack shape coefficient

## Abstract

The article presents research on the performance of different distributed fibre optic sensing (DFOS) tools, including both layered cables and monolithic composite sensors. The main need for the presented research was related to the growing applications of the DFOS techniques for the measurements of cracked concrete structures. There are no clear guidelines on the required parameters of the DFOS tools, which, despite their different designs, are offered for the same purpose (strain sensing). The state-of-the-art review and previous experiences show noticeable differences in the quality of the results depending on the applied DFOS tool. The technical construction of selected solutions was described with its theoretical consequences, and then laboratory tests on full-size reinforced concrete beams were discussed. Beams equipped with embedded tools were investigated in four-point bending tests, causing the formation of multiple cracks in the tension zone along the beams’ length. The results in the form of strain profiles registered by selected DFOS tools were analysed regarding the qualitative (crack detection) and quantitative (width estimation) crack assessment. The comparison between crack-induced strain profiles was based on a new parameter called crack shape coefficient CSC, which could be applied to assess the effectiveness of the particular DFOS tool in crack detection and analysis. It was one of the world’s first research allowing for such direct comparison between the layered and monolithic sensing tools. The summary indicates practical guidelines referring to the preferable design of the tools best suitable for crack measurements, as well as the field proofs based on data from two concrete bridges in Germany.

## 1. Introduction

The development of structural health monitoring (SHM) [1,2] allowed for a better understanding of the behaviour of the structures being operated in real environmental conditions under the influence of a wide range of impacts and time-depended effects [3]. Thanks to that, the concept of optimal decision-making [4] based on reliable measurement data could be put into practice. Usually, these decisions are focused on keeping the appropriate level of safety (acceptable risk) [5] while optimising financial savings. This simple rule is valid for both new structures (very often with complicated geometry, advanced construction technologies or new material solutions) and ageing ones (with an unknown technical condition or documentation), for which safe maintenance is of great importance [6]. It is worth underlying that failure consequences in civil engineering and geotechnical facilities include not only financial, social, and environmental losses but also loss of human health or life. This is the reason for the constant development of new solutions aimed at improving the structures’ long-term performance.

The better our knowledge (data), the better the structural assessment [7] and the better the decisions, especially taking into account the complexity of civil engineering projects. Usually, spot gauges measuring various physical quantities, such as strains, displacements, inclinations, accelerations, forces, and others, are installed within the structure or construction site to monitor changes in these values over time during variable loading and thermal conditions. A well-designed system should provide useful data for structural assessment, validation of simplified models, and, finally, decrease the risk.

This is the reason why new sensing technologies used in SHM are constantly being sought, and existing ones are being improved. The main limitation of conventional systems based on spot gauges is that they are not able to directly answer the question about the state of the structure between the measurement points.

**Figure 1:**
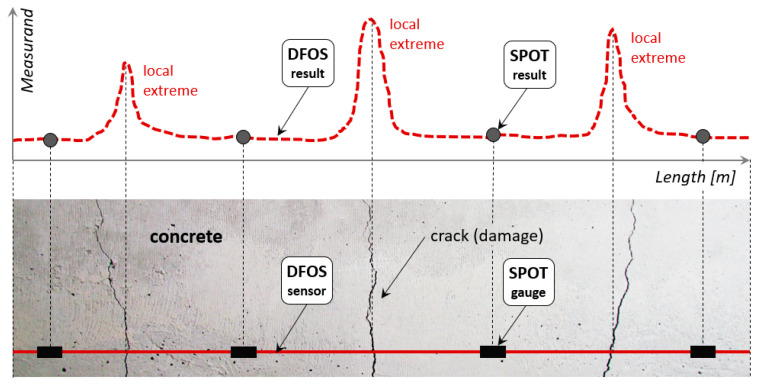
Graphical comparison between the capabilities of DFOS sensors and spot gauges.

The solution to this problem is proposed by distributed fibre optic sensing (DFOS), which, in contrast to spot gauges, allows the measurements to be performed continuously over the structural length (Figure 1). Therefore, there is no way to omit any extreme values, so one of the main goals of structural health monitoring—direct damage detection and its size estimation—can finally be fulfilled.

The ability for distributed sensing is the fundamental advantage of this technology, significantly influencing the growth of its applications (both laboratory and field) in recent years. Instead of a single number provided by the spot gauge, DFOS tools measure the profile of selected physical quantities over the entire structural length. That is why DFOS-based systems can be compared to the human nervous system [8,9], where sensors are nerves providing data, and the data logger is the brain for their post-processing and interpretation. The concept of smart structures with integrated DFOS sensors is more and more appreciated for measuring different quantities, including strains [10,11], cracks [12,13], displacements [14,15,16,17], temperatures [18,19], or vibrations [20]. Moreover, both static and dynamic readings [21,22] can be performed, which, for example, could be particularly useful for the real-time response analysis of the structure.

**Figure 2:**
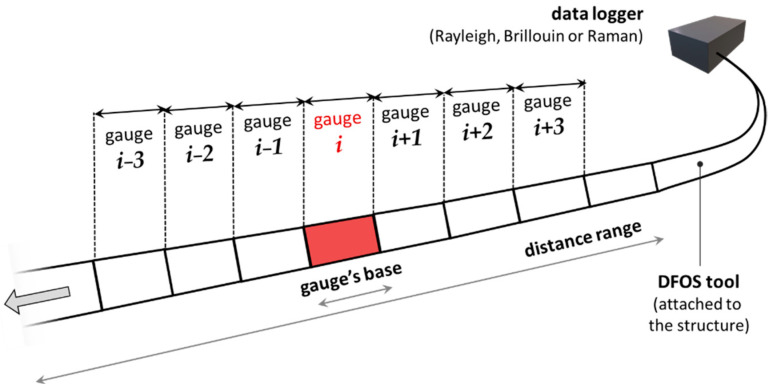
A simplified interpretation of spatial resolution in distributed measurements.

Currently, there are three main optical phenomena utilising the concept of distributed sensing: Rayleigh, Brillouin, and Raman scatterings. They have found their way into the design of optical data loggers available on the market. Each of them produces several DFOS sub-techniques characterised by individual advantages and limitations related to measurement parameters [12,23]. These are the type of the measurand, distance range, spatial resolution, accuracy, repeatability, measurand’s resolution, measurand’s maximum range, optical budget, acquisition time, operating temperature, and more. Spatial resolution is one of the critical parameters which could be understood, in simple terms, as the number of gauges per unit length of the DFOS tool. This can be visualised as a chain of spot strain gauges connected in series, in which the bases are adjacent (the end of one is the start of the next) (Figure 2).

The selection of the appropriate technique and data logger should always be preceded by the individual analysis of the specifications and requirements of a given project. However, certain generalities are outlined below. They do not constitute irrefutable rules, especially considering the dynamic development of DFOS-based devices:

1. **Rayleigh scattering** [22,24] measures both the mechanical and thermal strains. It provides high spatial resolution [21] (mm-order, meaning even more than 1000 gauges per 1 m of the DFOS tool). This is particularly useful for analysing localised events such as cracks or other structural discontinuities. However, the main restriction is usually the limited distance range (while maintaining high spatial resolution).
2. **Brillouin scattering** [25,26], similar to the Rayleigh, measures both the mechanical and thermal strains. However, it provides a lower spatial resolution (usually 20 to 100 cm). On the other hand, the readings can be implemented over very long distances (e.g., 25 km or more). This makes it a suitable solution for the global monitoring of linear structures such as pipelines, tunnels, roads, embankments, dams, or mining and landslide areas. It must be underlined that improved spatial resolution [27] in Brillouin sensing systems is feasible and consistently developed.
3. **Raman scattering** [28,29] is used to measure the temperature profile in optical fibre over long distances ranging from 25 km and more, while standard spatial resolution starts from 50 cm. Distributed temperature sensing (DTS) can be used as compensation for Rayleigh and Brillouin measurements or as an independent diagnostic solution for the thermo-detection of fires [30] or leakages.

The current opportunities also include reading the same DFOS tools using different DFOS techniques to obtain extended benefits. For example, automatic thermal compensation [31] or simultaneous measurements of various physical quantities are possible.

**Figure 3:**
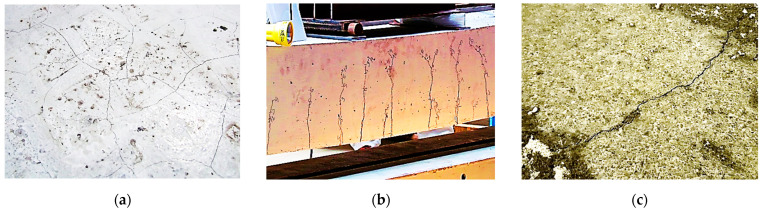
(**a**) Irregular thermal-shrinkage cracks within the surface of the slab; (**b**) multiple cracks within the tension zone of the bent beam; (**c**) single crack in the concrete road.

The article is focused on measurements within concrete structures. The most significant challenge for their diagnosis is their operation in the cracked state, which is a normal behaviour [32] started already after concreting [33]. Early-age concrete hydration causes thermal shrinkage strains, which are blocked by internal (reinforcement) and external constraints [34], finally causing the formation of cracks. They then develop under the influence of operating conditions, including mechanical loading and progressive structural degradation. The knowledge about the actual crack state is a fundament for assessing the technical condition and, thus, the structural safety. Figure 3 shows various crack states within different types of concrete structures. The cracks formed within the concrete structures usually have widths much lower than 1 mm, so they should be treated as a local event. This is the basis for selecting the DFOS technique for strain measurements with appropriate high spatial resolution.

**Figure 4:**
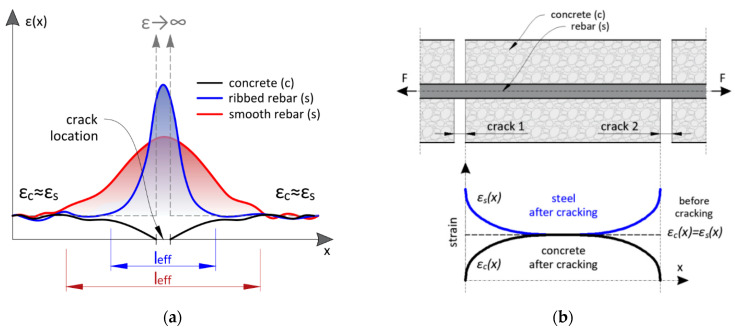
Strain distributions of concrete and steel rebar over length: (**a**) within the close vicinity of the crack; (**b**) within the area between the cracks.

Local discontinuities cause theoretical strains to tend to infinity within this area. The concrete disappears within the crack, influencing the strain profiles in reinforcing bars passing through the crack. By assuming the ideal bonding between these two materials, the rebar should also be broken, but this is not happening. The reason for that is debonding over some distance around the crack, called further the effective length *l_eff_*. This length depends on the material properties (both concrete and steel rebars) and bonding properties at the contact surface. For the rebars with the smooth outer surface, the crack-induced strain peak value will be lower, while *l_eff_* will be longer. On the other hand, the peak will be higher for ribbed rebar, while the *l_eff_* shorter (Figure 4a). By assuming that the cracks’ spacing is longer than the effective length, concrete and steel strains should be equal in the areas between the cracks (Figure 4b). In practice, cracks’ spacing depends on the strength and elasticity of concrete, static and loading scheme, boundary conditions, diameters, arrangement of the reinforcing bars inside the concrete, and many other factors.

The general scheme of the DFOS tool’s operation within the crack area is similar to the reinforcing bars. The accumulation of strains over a very short distance causes the strain peak to be somehow distributed over a few to several dozen centimetres, depending on the type of concrete and the quality of the DFOS tool. Despite the crack width or its change reaching only the tenths of a millimetre, the spatial resolution of mm or cm order is sufficient to analyse the above phenomena correctly.

**Figure 5:**
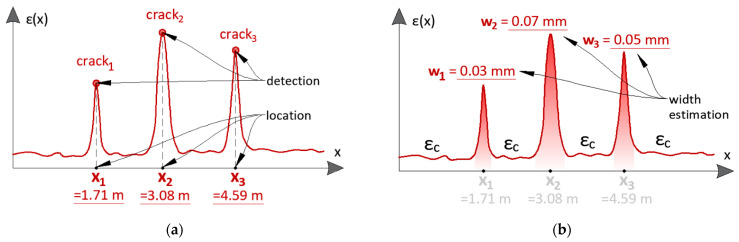
The concept of the DFOS system goals in cracked concrete structures: (**a**) qualitative analysis (crack detection and location); (**b**) quantitative analysis (crack width estimation, strain measurements between the cracks).

The main goals of distributed sensing for cracked concrete assessment are summarised in Figure 5. Firstly, the detection of cracks must be possible. In other words, the crack-induced strain peaks must be sharp enough to identify the crack presence clearly. Next, based on the installation documentation, the locations of cracks must be specified in relation to the structure’s geometry. These two steps are further called qualitative analysis (Figure 5a). On the other side, we have the quantitative analysis to estimate the cracks’ widths with sufficient accuracy, meaning better than 0.05 mm from an engineering point of view (Figure 5b). In cases of non-cracked concrete (e.g., with regard to prestressed structures) or when the crack spacing is greater than *l_eff_*, the sensor’s task is to reflect the actual structural strains accurately. However, it must be underlined that within the debonding area, this goal is transformed into the correct detection and estimation of the damage size instead of analysing the concrete deformations as a continuous material.

The ability to detect cracks by distributed fibre optic sensing was reported in many papers [35,36,37]. This ability is not in doubt, but it should not be treated unconditionally. The possible situations when poor-quality data can be misinterpreted are demonstrated and discussed hereafter. Similarly, the literature [38,39,40,41] describes different approaches for crack width estimation, considering the applied spatial resolution and assumed physical model of the entire system (DFOS tool (installation method) concrete). In the simplest terms, such calculations may rely on summing the strains over the effective length, which gives conservative results, slightly overestimating the actual crack widths.

Another important aspect of data interpretation is the installation method, which influences the system’s reliability and efficiency. For the existing, ageing infrastructure, the common approach is to glue the DFOS tools on the prepared (ground, cleaned, and degreased) surface or inside the pre-made near-to-surface grooves [42].

However, the article focuses on new structures where it is possible to place (embed) tools inside the concrete [43,44,45]. This is the most convenient way and, at the same time, the most favourable in terms of interpretation. The benefits include:

1. The possibility of analysis of the structural behaviour from a real zero stress–strain state through all the important phases [46], such as early-age concrete [47], thermal-shrinkage strains and corresponding micro-cracks [48], prestressing [49], construction stages, load tests [17], and, finally, regular operation. Structural assessment can be performed based on the absolute values of measured parameters (strains or crack widths) instead of their changes (increments). When installing on the existing structures, the initial level of stress and deformation is unknown or estimated theoretically with significant uncertainties.
2. The best possible integration of a particular DFOS tool with the concrete, as it thoroughly covers the outer surface of the tool. It ensures the best possible strain transfer from the structure to the sensor with no additional mounting brackets or elements.
3. Natural protection of the embedded tools against mechanical damages, aggressive external environment, or direct sunlight. The predicted operation lifetime of such a DFOS system is comparable with the operation lifetime of the structure itself.

**Figure 6:**
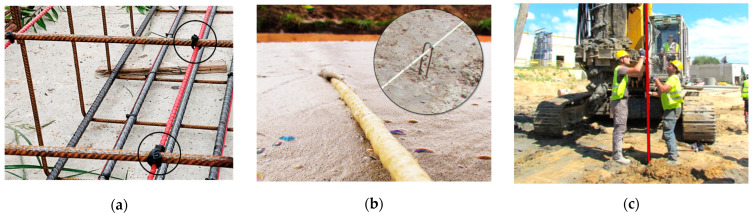
Possibilities of the sensors’ embedding methods: (**a**) tying to the existing reinforcement; (**b**) using special mounting brackets in the foam concrete without reinforcement; (**c**) application of advanced system in a concrete column without reinforcement.

When a structural element is reinforced, which is the most common situation in civil engineering projects, the DFOS tools can be tied to the rebars using cable ties (Figure 6a). Usually, sensors are cut at the required length, delivered on-site in coils, unrolled and stabilised in designed positions. However, the applications are not limited only to reinforced elements. Figure 6b presents an example of the foamed concrete layer within the road structure, where DFOS tools were installed using special mounting brackets. Another case in point concerns concrete columns, where a special installation procedure was elaborated and applied in order to embed the sensors [50]. A steel tube with the sensors ended with an anchor was pressed into the concrete; the anchor was then relieved, and the tube was pulled outwards, leaving the sensor inside.

One of the primary goals of the research presented hereafter and, at the same time, its novelty is the direct comparison of the indications of selected, commercially available DFOS tools embedded in the same structural beam working in a cracked state. Based on that, the set of features most desirable for the efficiency of cracks’ analysis was identified.

## 2. Review of DFOS-Based Tools

### 2.1. General Classification and Overview

The entire DFOS-based system consists of data loggers based on selected scattering and the DFOS tools attached to the concrete. That is why optical devices are usually designed by physicists, optical and electronics engineers, and IT specialists, while DFOS tools should also involve civil engineers, materials scientists, and mechanics.

There are three main groups of sensing tools [12]:

1. Optical fibres in their primary coating;
2. Layered sensing cables, where optical fibre is protected by a set of outer layers (coatings, covers, jackets, or strengthening inserts);
3. Monolithic sensors, where fibre is integrated inside a single-material core (usually composite).

**Figure 7:**
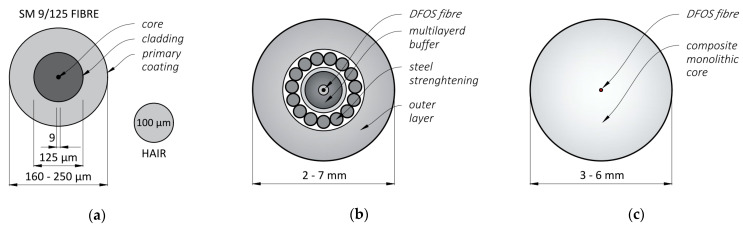
Example cross-sections of (**a**) single-mode optical fibre in its primary coating, (**b**) layered sensing cable with steel strengthening insert, and (**c**) monolithic strain sensor.

The example cross-sections of the above tools are presented in Figure 7a–c, respectively. Due to the small size of the standard fibres (outer diameter up to 250 µm), the first group has found their application primarily in the laboratory, where installation conditions can be precisely supervised. Although the fibres are relatively resistant to longitudinal deformations along their axis, they are susceptible to transverse forces and can be broken easily. A very important aspect while applying the fibre as a DFOS tool is the type of its primary coating, which influences the maximum strain range, strain transfer mechanism or resistance to an alkaline concrete environment. In practice, the softer acrylate and stiffer polyimide coatings are most often used. The comparison between their mechanical response while attached to the structural surface [51] shows that polyimide provides a more precise strain transfer mechanism, reducing the length of strain mobilisation. They are usually a good solution for small-size laboratory specimens. The acrylate coatings are less preferable for short measurement distances, but on the other hand, they decrease the crack-induced strain peaks, thus minimising the risk of damage (breakage) to the fibre. It is worth noting that the final performance of the surface-bonded DFOS system is also strongly dependent on the parameters of the adhesive [39,52], which could be treated as an additional layer around the fibre in the primary coating. Therefore, the effectiveness of any solution should be analysed, taking into account all its items and the interactions between them. Another aspect related to the coating type is its durability and resistance to alkaline concrete environments. Paper [53] proves that polyimide coating degrades after just 14 days of the alkaline attack, while no significant influence is observed on the acrylate one. Polyimide coatings without additional protection jackets are therefore not recommended for long-term measurements of concrete structures.

Although the systematic, safe handling of the fibres in their primary coatings on site is not feasible due to the high failure risk, they are the in-built measuring elements in other DFOS tools. These are layered cables and monolithic sensors widely used in civil engineering [54,55] and geotechnical [56] applications, including bridges [57], roads [58], collectors [59], pipelines [60,61], and many others. A brief summary of each of these two different concepts is presented in the following subsections.

**Figure 8:**
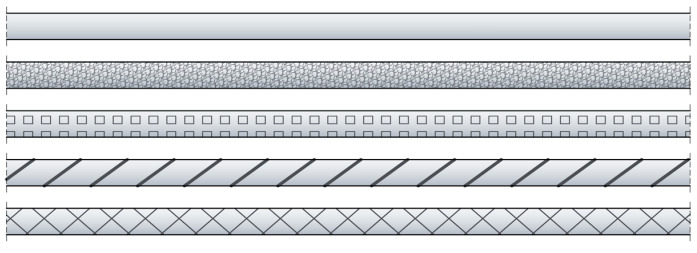
Example ideas for the outer surface of the DFOS tool (from top): smooth, with sand grains, with perforated groves, with unidirectional thick braid, with bidirectional thin braid.

Despite the noticeable differences in the design of layered cables and monolithic sensors, the same requirements should be placed on them for monitoring cracked concrete. First of all, they must provide high accuracy ensured by the best possible strain transfer from the host material (concrete) to the measuring fibre inside. For example, a rough outer surface can increase the bonding properties between the concrete and the DFOS tool, supporting the adhesion by mechanical clamping (such as ribs in reinforcing bars). Instead of a smooth surface, additional roughening may be achieved by sand grains, perforated grooves, or external braids. The examples of ideas of possible outer surfaces are summarised in Figure 8.

On the other hand, the strain transfer mechanism can also be improved by a tight connection between all the components inside the tool, eliminating possible slippage.

The next requirement is the high-strain range, as local crack-induced strain peaks can achieve even 30,000 µε or more [62,63]. The DFOS tools should withstand such conditions without the fear of breakage. That is why the materials used to build a given tool should be characterised by a wide strain range. For example, plastic or steel layers yield approximately 2000 µε, clamping around the fibre and preventing it from correct measurements of the actual state of the structure. This effect is especially worrying during long-term measurements of cracks suspected to the cyclic changes in their widths.

Resistance to harsh conditions during installation and operation is the next issue that should be considered. It includes resistance to mechanical damages, local transverse forces (e.g., the pressure of aggregate grains or mounting elements), alkaline concrete environment, and other aggressive chemical impacts. The expected operational lifetime of the DFOS tools embedded inside the concrete is comparable to the structure’s lifetime. That is why the appropriate durability [64] should characterise them.

Finally, the mechanical parameters of the DFOS tool (including diameter and stiffness) should facilitate the installation process, for example, ensure correct positioning without extensive waving. Any deviations from the designed position will result in additional errors during data interpretation. It should be possible to achieve straight sections and reliable readings both in compression and tension zone without the need for pre-tensioning, which, on a construction site, is very often difficult or even infeasible.

### 2.2. Layered Sensing Cables

Both layered cables and monolithic sensors are aimed to enable DFOS measurements within the construction or field conditions, including the embedding of the fibre inside the concrete. However, their internal design involves two different technical solutions.

**Figure 9:**
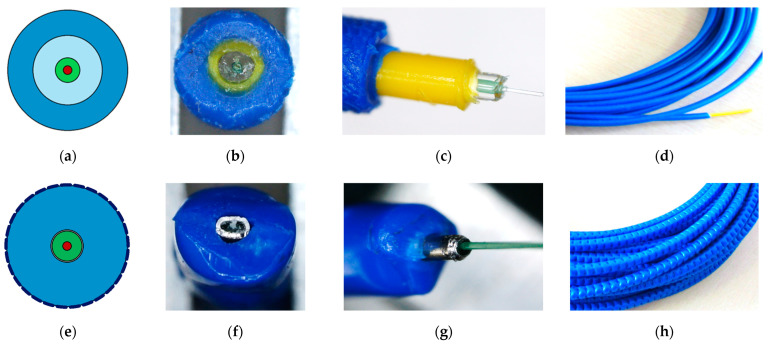
Example layered cables C1 and C2, respectively: (**a**,**e**) design; (**b**,**f**) cross-section; (**c**,**g**) side view with particular layers; (**d**,**h**) the view of outer surface.

In the first group, the optical fibre is protected by a set of outer layers with different physico-mechanical parameters, in some way connected to each other. This solution is also known for a wide range of telecom applications. The layers provide protection against both mechanical and chemical impacts on the internal fibre or fibres. Usually, the layers are made of plastics such as polyamide (PA) or high-density polyethylene (HDPE). In some designs, there are additional steel inserts aimed at improving the strength parameters of the entire cable. Figure 9 presents the two commonly used layered cables, which were also investigated during the research described hereafter. The following notations have been adopted for further analysis in this article:

1. C1—sensing cable with two plastic layers, steel insert, and a perforated outer surface;
2. C2—sensing cable with three plastic layers and a smooth outer surface.

Internal design, cross-section, side view, and the outer surface of the solutions are presented in Figure 9a and Figure 9e, Figure 9b and Figure 9f, Figure 9c and Figure 9g, and Figure 9d and Figure 9h, respectively.

**Figure 10:**
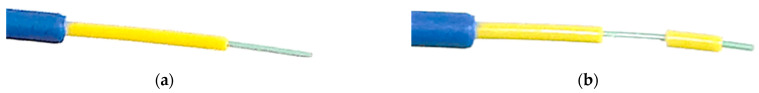
Graphical presentation of possible slippage between the layers: (**a**) stripped three-layer cable; (**b**) the piece of intermediate layer slipped against the internal layer.

While performing structural measurements, the presence of each layer should be justified. For example, a perforated outer surface is aimed to improve bonding properties with the surrounding concrete. The original concept of layered design and its substantive justification was presented in patents [65,66] and further publications [67,68]. The presence of layers influences the strain transfer [69,70], reducing the high peaks and, thus, the risk of fibre breakage. This effect is caused by the geometry, material properties and possible slippage between layers. Figure 10a shows the stripped three-layer cable, while Figure 10b shows the possible slippage between the intermediate and internal layers. Such an effect can uncontrollably increase the effective length in the DFOS-based crack analysis.

**Figure 11:**
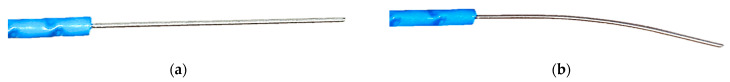
Layered cable with steel strengthening insert: (**a**) before the deformation; (**b**) after the permanent (plastic) deformation.

The higher the strains, the higher the probability of slippage. On the other hand, elastic–plastic metal coatings are able to deform permanently. For example, the yielding point of steel is equal to only 0.2%, while crack-induced strain peaks exceed this value multiple times. During intense tension, according to Poisson’s law, they also clamp around the fibre. Finally, the strains along the fibre can be kept even after removing the force (closing the cracks). Figure 11a shows the layered cable with steel insert before deformation, while Figure 11b shows the cable deformed in a permanent (plastic) way.

Thus, the initial concept of applying the additional layers around the optical fibre, apart from its mechanical protection, allowed for achieving the event memory. Even after unloading the structure, well-designed cable allowed measuring historical strains, indicating the most strained, safety-critical areas. It was useful, for example, when analysing the spacecraft’s performance. Instead of continuous monitoring during the flight, the measurements could be performed after its return to the earth.

The above ability indicates the need for a custom design of layered cable depending on the application and predicted strain values within the structure in question. Selection of the layers’ number, their mechanical parameters (the material), and geometry (thickness) should be performed with caution because it will influence the interpretation of measured strains, which will differ from the actual structural strains. The literature, for example [71,72,73,74], provides a number of advanced mathematical–physical models and equations to describe strain transfer through a set of layers with different parameters. In order to align the results to the observations as well as possible, some of them must consider interlayer slip and the post-yielding behaviour of the materials. Usually, such models produce significant uncertainties and are challenging to apply in practice for analysing individual cracks suspected of cyclic load changes.

In addition to the mechanical strain analysis, thermal influences must also be considered. Different thermal expansion coefficients of subsequent layers and different connection characteristics may produce unknown mechanical strains in the measurement fibre inside the cable. Thus, the properties of the cable’s components and their adhesion parameters must be carefully studied. Usually, it is recommended to calibrate the response of a particular cable in the thermal chamber, where both mechanical strains and external temperature can be controlled.

Another feature of the discussed solution is suggested by the name “*cable*”, which, for example, in the finite element nomenclature, refers to the element transferring only the tension forces. Thus, in some cases, pre-tensioning the sensing cables is needed during installation [73] if compression strains are expected during operation. This process also makes it easier to keep the DFOS route straight without extensive waving. However, it may be challenging or sometimes impossible under construction or field conditions.

Sensing cables are widely used to monitor linear structures or geotechnical facilities over long distances. Usually, they are connected to Brillouin or Raman-based data loggers, with spatial resolution in the range of 50–100 cm, enabling global qualitative analysis (e.g., detection of fire or leakage). As they are designed for strain measurements, they are also used for structural analysis of concrete structures. Thus, their performance with high spatial resolution Rayleigh-based devices will be investigated further compared to other types of DFOS tools. The local analysis of cracks will be of the main interest.

### 2.3. Monolithic Sensors

**Figure 12:**
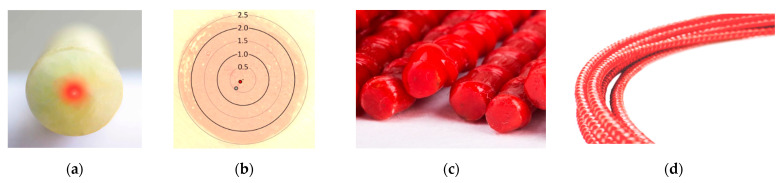
Example of stiff monolithic DFOS strain sensor: (**a**) cross-section; (**b**) microscopic view with the scale and marked optical fibres; (**c**) side view; (**d**) the view of the outer surface.

Another concept for providing fibre protection for field applications was introduced by monolithic sensors. The technology known for the production of composite elements, called pultrusion [74], was used for that purpose. Instead of multiple layers surrounding the optical fibre, it is fully integrated with a monolithic core. Fibre-reinforced polymers (FRP) consist of fibres (reinforcing elements) connected together by epoxy (matrix). The heating process in a thermal chamber supports the epoxy hardening to achieve the required mechanical parameters. During that process, optical fibre with similar diameters and properties, such as reinforcing fibres, is used as one of them. Thus, it is fully integrated within the monolithic cross-section (Figure 12a). Any possible disturbances caused by the internal slippage within the DFOS tool are then minimised, reducing uncertainties and simplifying mathematical models used for strain transfer analysis.

Figure 12b shows the microscopic view of the cross-section of the bar with a 5 mm diameter with marked locations of two optical fibres integrated during pultrusion. Additional fibre can be used as a redundant or for simultaneous measurements using different data loggers. The authors have experience with combined distributed sensing of strains and temperatures (DSS + DTS), as well as strains and vibration (DTS + DAS). Thanks to that hybrid approach, extensive knowledge of structural performance can be obtained while minimising the installation efforts.

Monolithic sensors involve the technology of FRP bars, which are increasingly used to reinforce concrete structures [75] and thus must be complied with the relevant standards in terms of strength, elasticity and bonding properties. Usually, outer unidirectional or bi-directional braids are used (Figure 12c,d) to provide good interaction with surrounding concrete (not only by adhesion but also by mechanical clamping). Monolithic sensors can therefore replace the composite bars and be included in strength calculations of the structure itself. In other words, they could have a double function, both sensing and reinforcing at the same time. It is worth underlying that the application of FRP reinforcement is also favourable from a durability point of view [76], as it is fully resistant to corrosion. That aspect should be considered for long-term structural health monitoring, especially when DFOS tools are operated in harsh environmental conditions.

**Figure 13:**
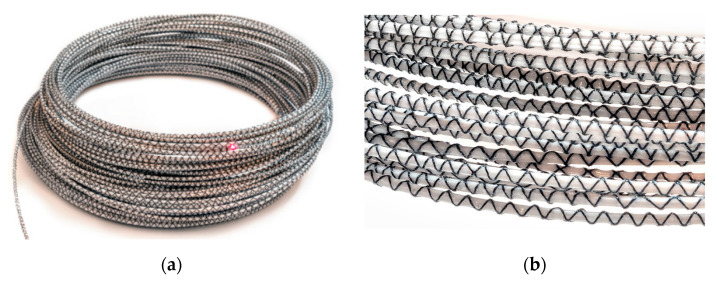
Example of flexible monolithic DFOS strain sensor: (**a**) general view; (**b**) close-up to the bi-directional outer braid.

Although the general design of monolithic sensors is always the same, their material and geometric properties could be adjustable depending on the specific requirements. For example, for small-size laboratory specimens, the application of a stiff sensor is undesirable because it changes the behaviour of the concrete specimen in question. In order to avoid local strengthening of the concrete, it is possible to decrease the sensor’s diameter and elastic modulus. For example, glass reinforcing fibres can be replaced with polyester together with a special type of epoxy matrix. For the stiff sensor presented in Figure 12, the elasticity modulus is 50 GPa, the diameter is 5 mm, and the maximum strain is ±2% (±20,000 µε). Figure 13a show the flexible version after modifications, with an elasticity modulus of 3 GPa, a diameter of 3 mm, and a maximum strain of ±4% (±40,000 µε). This means a 50 times reduction in axial stiffness, making the sensor invisible to the concrete. Figure 13b presents the close-up of the outer bi-directional braid of that sensor. It is worth noting that the geometry of the cross-section can also be modified. For example, for surface gluing applications, the circle section can be replaced with a flat rectangle, and outer braid can be removed.

Despite the significant reduction in stiffness, it is still not so low as to make installation problematic through excessive waving or the need for pre-tensioning. Monolithic sensors work in both tension and compression, provided that they are protected against buckling. This problem is naturally solved while embedding inside the concrete.

The research presented hereafter involves two types of layered cables embedded inside the concrete beams together with two types of monolithic sensors (stiff and flexible versions). The following notations have been adopted for further analysis in this article:

1. M1—monolithic, reinforcing sensor (Ø5.0 mm, E = 50 GPa);
2. M2—monolithic, flexible sensor (Ø3.0 mm, E = 3 GPa).

### 2.4. Monolithic Sensors and Layered Cables—Summary

**Figure 14:**
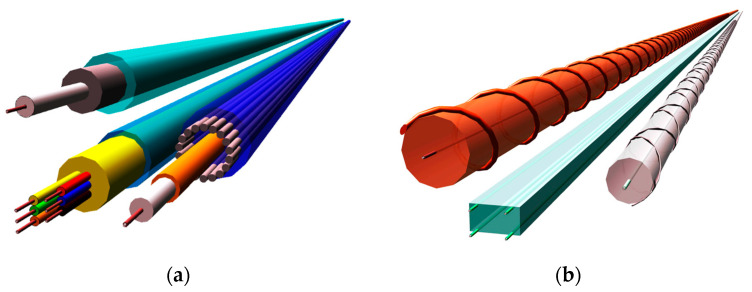
Spatial visualisation of example (**a**) layered cables; (**b**) monolithic sensors.

The review of DFOS tools available on the market indicates that their technical design differs significantly. Figure 14 shows the spatial visualisation of selected examples of layered cables (Figure 14a) and monolithic sensors (Figure 14b). It should be remembered that the initial concepts of these two solutions were intended for different objectives. Even though they are currently very often used for the same purposes (e.g., strain sensing), there is a concern that the obtained results will be considerably different.

Table 1 summarises the general features of layered sensing cables and monolithic sensors based on the state-of-the-art review and the authors’ own previous experiences in laboratory and field applications. It should be underlined, though, that this is just overall information usually corresponding to the proposed classification, but a detailed analysis should always be carried out for the individual model of the sensor or cable.

## 3. Laboratory Tests

### 3.1. The Concept and Preparation of the Specimens

**Figure 15:**
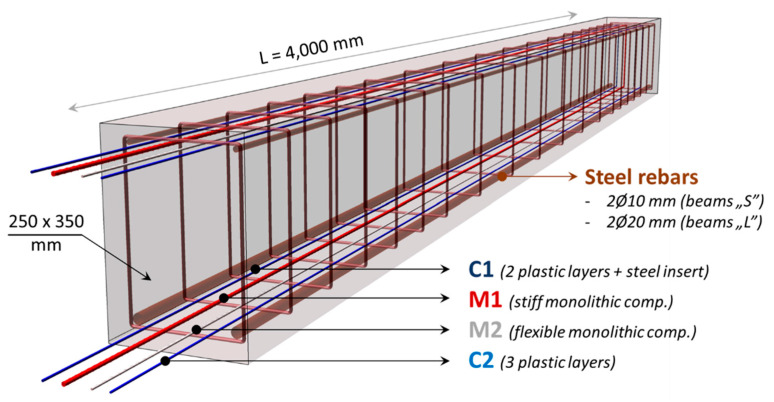
Spatial visualisation of the concrete beams equipped with embedded DFOS tools.

The research described hereafter was aimed at directly comparing four DFOS tools embedded in the same reinforced concrete members. It was one of the first attempts in the world to use commercially available layered cables and monolithic sensors arranged parallelly to monitor structural strains and cracks. A total of six beams with a rectangular cross-section of 250 × 350 mm and a length equal to 4 m were made, equipped with two sensing cables (C1 and C2; see Section 2.2) and two monolithic sensors (M1 and M2; see Section 2.3). The tools were installed both in the compression (upper) and tension (lower) zone of the beams. The spatial visualisation of the specimen is presented in Figure 15. Beams were differentiated by the diameters of the main reinforcing bars. Three beams, called “S”, have smaller diameters (2 × Ø10 mm), while the next three, called “L”, have larger (2 × Ø20 mm). The aim of that approach was to obtain different crack patterns, both in terms of their widths and spacings. For L-type beams, more cracks were obtained with smaller spacings and widths. On the other hand, for S-type beams, fewer cracks with greater spacings and widths were obtained.

**Figure 16:**
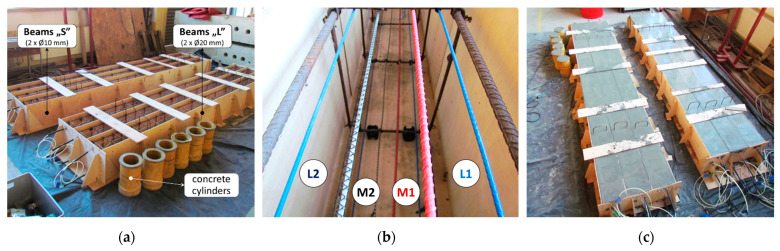
(**a**) View of formwork prepared for concreting; (**b**) close-up to the DFOS tools; (**c**) the view of the beams after concreting.

The beams were concreted using two formworks (Figure 16a). Moreover, six concrete cylinders were made to verify material properties (strength and elastic modulus) in a standard compression test. All the tools were stabilised parallelly (Figure 16b) by tying them to the steel stirrups. During the installation, attention was paid to the various resistance of each tool to point pressure and to keeping a straight designed path without excessive waving. In this context, the L2 cable proved to be the worst and the M1 sensor the best. However, with special care and attention, all the tools could be installed successfully.

Because of the risk of shifting densely spaced tools during concreting and vibrating, it was decided to use self-compacting concrete. After concreting, the top surfaces of the beams were left without any protection against drying—no curing procedure was followed. The idea behind that was to intensify the thermal-shrinkage strains of early-age concrete and, thus, the formation of microcracks. This process was supported by using concrete with a large amount of Portland cement. As a result, it was possible to analyse not only the new cracks formed in the tensile (lower) zone during mechanical bending but also the closing of the existing shrinkage cracks in the compression (upper) zone.

Table 2 summarises the technical specifications of all the DFOS tools used during the research. From the crack detection and analysis point of view, the most important are strain resolution and range, diameter, axial stiffness (as a product of cross-section area and elastic modulus), internal design, and outer surface.

### 3.2. Course of the Measurements

**Figure 17:**
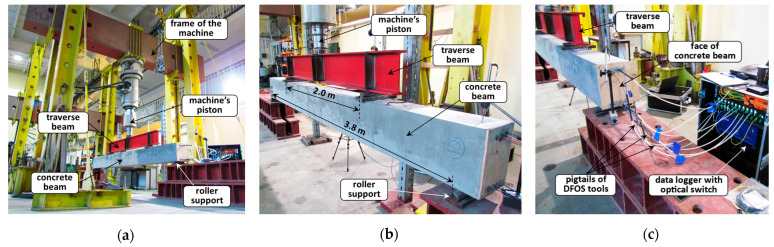
(**a**) The view of the measurement station; (**b**) close-up to the example beam; (**c**) Rayleigh-based data logger with an optical switch connected to embedded DFOS tools.

The mechanical four-point bending tests of all the prepared beams took place in Building Materials and Structures Research Laboratory at Cracow University of Technology, Poland. The universal testing machine with the maximum load cell capacity of 1000 kN was used for that purpose (Figure 17a). The machine’s accuracy is ±0.5% of the reading down to 1/500 of the full scale, while the maximum force applied during research was equal to 210 kN. The beams were supported by roller supports with free rotations and horizontal movements while loaded through the traverse beam (Figure 17b).

The DFOS measurements were performed using a high-spatial-resolution, Rayleigh-based device witan h optical switch, allowing for simultaneous connection of all the tools embedded inside the beam (Figure 17c). The readings were performed in a static way while the force was fixed at selected levels.

**Figure 18:**
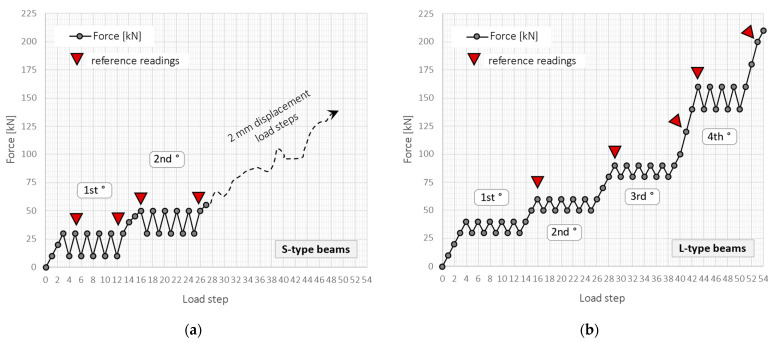
The loading schedule for (**a**) S-type and (**b**) L-type beams.

The loading procedure was designed separately for S- and L-type beams (Figure 18a,b) due to their different reinforcement and resulting different stiffness and strength (load capacity). Grey dots represented the load steps when DFOS measurements were taken, while red triangles correspond to the reference readings of crack widths on the side surface of the beam.

**Figure 19:**
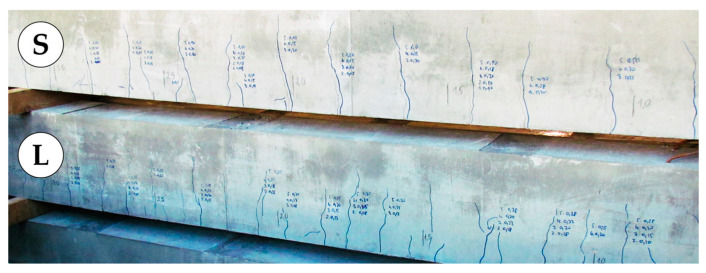
Documented crack width and spacings, for example, S- and L-type beam.

All the beams were loaded up to the structural failure, which was achieved in a different way: by losing the bending capacity (“S” beams) or shearing capacity (“L” beams). The obtained crack patterns were also different, according to the theoretical predictions. More cracks with lower spacings and widths were obtained for larger diameters of reinforcing bars, while for smaller rebars’ diameters, the situation was reversed (Figure 19).

### 3.3. Crack Documentation

Because of embedding the DFOS tools inside the concrete beam at the height of the main reinforcing bars, there is no technical possibility for directly comparing the measured width of individual cracks with other surface-based techniques. The course of the crack over the beam’s width is random. The authors are aware of this limitation. However, taking into account the statistical approach and analysis of multiple cracks, such a reference could be useful, giving a general overview of the crack state observed from the outside.

**Figure 20:**
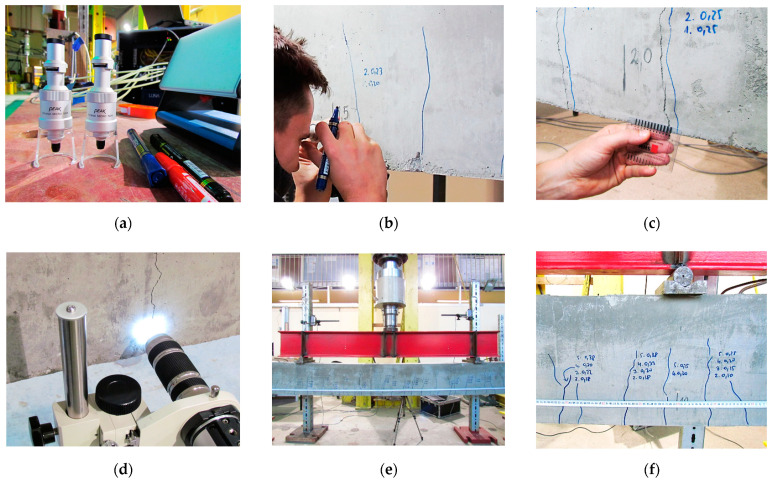
Crack documentation: (**a**) portable peak stand microscopes; (**b**) width measurement by optical microscope; (**c**) width measurement by plate crackmeter; (**d**) width measurement by digital microscope; (**e**) localising the cracks by metric tape; (**f**) close-up to the multiple cracks.

That is why during research, special attention was paid to documenting crack widths in selected load steps, as well as cracks’ locations over the length. Portable optical microscopes with a resolution of 0.02 mm were used to investigate small cracks (widths up to 0.4 mm) during the initial load steps (Figure 20a). An in-built scale above the focusing ring allowed for convenient measurements of widths along the side surface, on which the results were systematically written using a marker (Figure 20b). After the crack widths exceeded 0.4 mm, the plate crack meters with a resolution of 0.1 mm were used (Figure 20c). These simple indicators are made of transparent plastic with a printed scale.

Moreover, a digital microscope (Figure 20d) was used to monitor the selected midspan crack by measuring the distance between two defined points (indicated by the crack edges).

The final step in cracks documentation was focused on their locations. After the structural failure of each of the beams, all the cracks were highlighted with a marker. The metric tape was stretched all the way along the bottom edge of the beam, and detailed photo documentation was performed (Figure 20e,f). This approach allowed us to determine the location of cracks, counting from the face of the element, with an accuracy of 1 mm.

## 4. Results and Discussion

### 4.1. Tension Zone—Global Analysis of Strain Profiles

The research included the strain measurements during the early-age concrete state and the corresponding development of the microcracks. Furthermore, these cracks formed in the upper part of the beams were investigated further during mechanical bending tests. As, according to the applied static and loading scheme, they are located in the compression zone, it was possible to analyse their behaviour while closing. The example analysis and figures showing the strain profiles in the compression zone for the S-type beam were presented and discussed in the paper [77]. Although the results provided by all the analysed DFOS tools were satisfying, it should be underlined that the measured compression strains, resulting from the closing of the cracks, did not exceed 600 µε (much less than the declared maximum strains for particular tools; Table 2). The crack width changes were calculated by all the tools with the maximum differences not higher than 0.01 mm.

**Figure 21:**
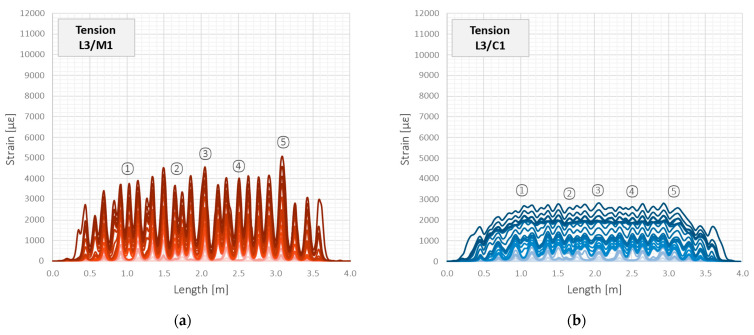

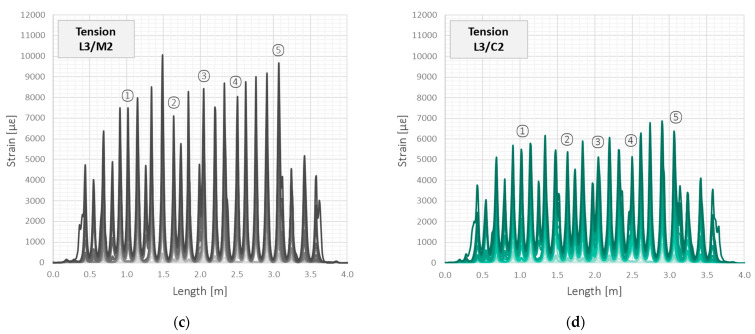
Beam L3/tension zone: strain profiles over entire length registered by the following DFOS tools: (**a**) M1; (**b**) C1; (**c**) M2; (**d**) C2.

However, while analysing the behaviour of reinforced concrete structures, the tension zone is of the main interest. That is why a detailed analysis of the tension zone of beam L3 will be investigated hereafter. Figure 21 shows the strain profiles over the entire structural length during subsequent load steps, corresponding to those presented in Figure 18b. The scale of the horizontal and vertical axes is the same for all the DFOS tools to facilitate visual comparison. Five strain peaks corresponding to the formed cracks were chosen for further statistical analysis and marked on the plots.

For the load step no. 53 and the corresponding force of 200 kN, the maximum strain value within the crack-induced area registered by the flexible monolithic sensor M2 was equal to 10,053 µε. For the cable C2, sensor M1, and cable C1, it was 6852 µε, 5080 µε, and 2832 µε, respectively. It means 68.2%, 50.5%, and 28.2% of the maximum value for M2. However, max. strain measured by different tools was not induced by the same cracks. An example parameter for global comparison of tensile strain profile could be a variation coefficient, which expresses the ratio of the standard deviation to the mean value (Equation (1)).

(1) $$V=\frac{stdv_{L}}{\varepsilon_{m,\;\;L}}=\frac{1}{\varepsilon_{m,L}}\cdot \sqrt{\frac{\sum_{i=1}^{K=L/sr}\;{\left(\varepsilon_{i}-\varepsilon_{m,L}\right)}^{2}}{K-1}}$$

where *V*—variation coefficient of strains profile (-); *ε_m,L_*—mean strain over the length (µε); *stdv_L_*—standard deviation of strains over the length (µε); *sr*—spatial resolution (equal to 10 mm); *L*—analysed length (4000 mm); and *ε_i_*—local strain in location *i*.

Statistical parameters calculated over the entire length for all the DFOS tools are summarised in Table 3 for selected load steps. These parameters include the mean and maximum strain value, standard deviation, and variation coefficient according to Equation (1). Assuming the spatial resolution of 10 mm and the length of the beam 4000 mm, a total of 400 samples for each tool were obtained for statistical calculations. Moreover, the mean values of each parameter were calculated and normalised to unity, which facilitates the comparison between the individual tools. Although the mean strain values are practically qual, which is a natural consequence of applying the same load conditions for all the tools embedded inside the same beam, the strain distributions over their lengths are significantly different. It can be concluded that the higher the variation, the better the measurement quality of cracked concrete and, thus, the better the tool for crack detection and analysis.

The most variable distribution was noted for flexible monolithic sensor M2. The reason for that was low axial stiffness and good bonding properties within the surrounding concrete. The second place was assigned to the flexible C1, which, despite its layered design, did not suffer significant internal slippage. However, there were most problems with it during installation, including extensive waving and no resistance to local pressures. In the third place was monolithic sensor M1, which, due to its high axial stiffness, acted as a local reinforcement for the concrete, reducing the actual crack width in its close vicinity while increasing the effective length.

**Figure 22:**
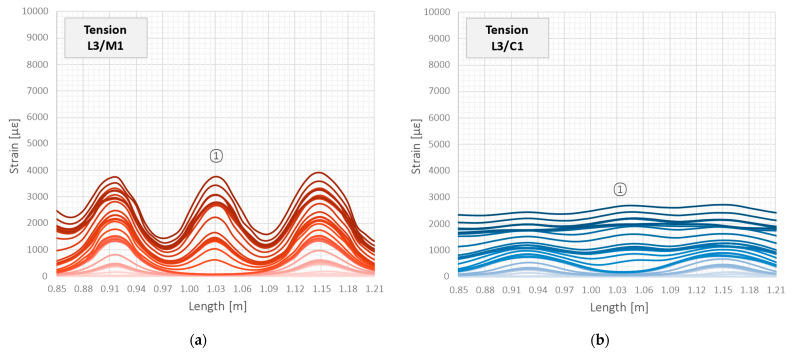

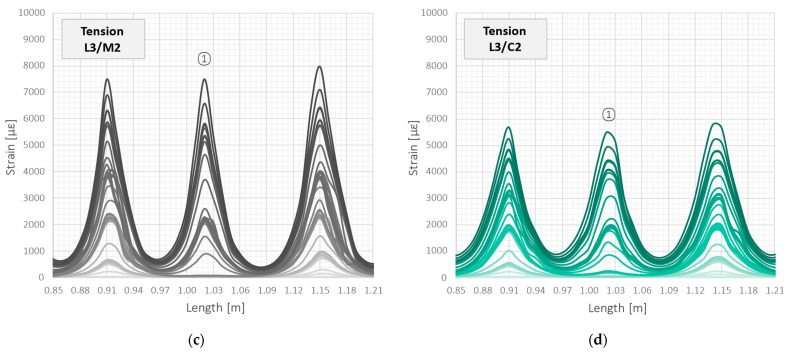
Beam L3/tension zone: strain profiles in close vicinity of crack no. 1 registered by the following DFOS tools: (**a**) M1; (**b**) C1; (**c**) M2; (**d**) C2.

The tools M1, M2, and C2 allowed for the unambiguous identification of cracks and their analysis throughout the entire research. However, the results for cable C1 with steel reinforcing insert were not acceptable. Extensive internal slippage was observed, which averaged the strains along the length in such a way that precise detection of the cracks was no longer possible. Figure 22 shows the close-up of the strain profiles registered within the close vicinity of crack no. 1 in the subsequent load steps. For cable C1, the very smooth plots pose a hazardous risk of not detecting a crack by the DFOS system, which is unacceptable during the measurement-based structural assessment. This is because crack detection is one of the main goals and objectives of such analysis.

**Figure 23:**
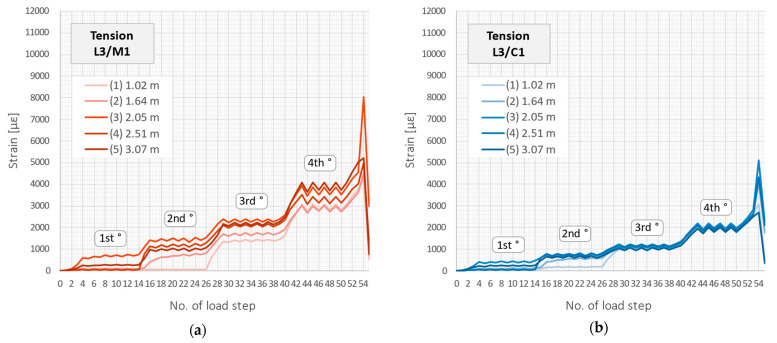

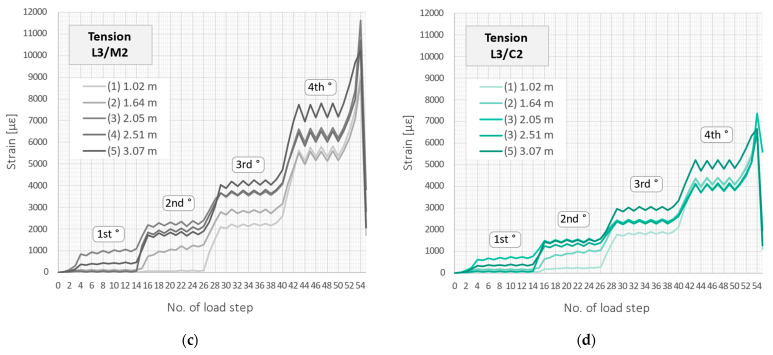
Beam L3/tension zone: crack-induced strain peaks over subsequent load steps registered by the following DFOS tools: (**a**) M1; (**b**) C1; (**c**) M2; (**d**) C2.

The above observation about a significant decrease in the crack-induced strain peaks and averaging the strains over length for cable C1, and correct behaviour of other tools, was valid through all the load steps. However, the effect becomes more visible under the higher load values in the second, third, and fourth load cycles. Figure 23 shows the maximum strain values in subsequent load steps for the five selected cracks, which locations are marked in Figure 21. For all the tools, excluding cable C1, the differences between these values were kept during the entire research, which is natural and corresponds to the different widths of these cracks. However, by analysing the behaviour of cable C1 (Figure 23b), it can be seen that the values for the five cracks at different locations are very similar. This is because the measurement fibre inside the cable slips inside the layers and does not reflect the actual deformation state of the structure. Even assuming that the actual cracks’ locations are known by external observation, calculations of their widths based on that strain profile are subjected to high uncertainties.

**Figure 24:**
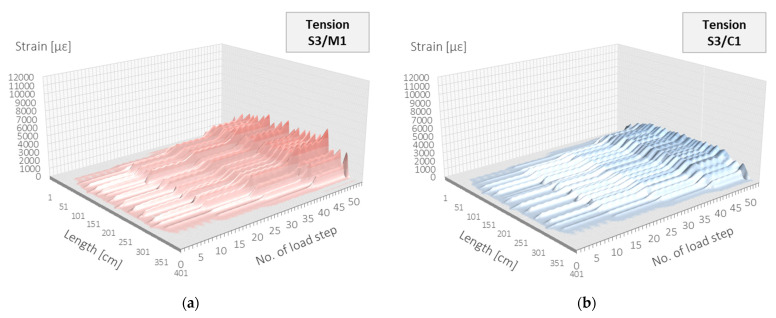

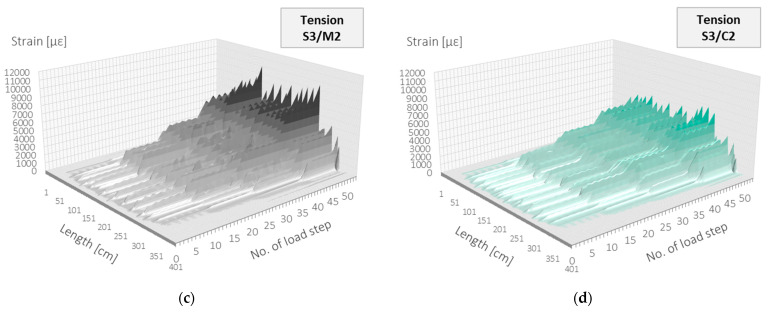
Beam L3/tension zone: strain profiles in length and load step domain registered by the following DFOS tools: (**a**) M1; (**b**) C1; (**c**) M2; (**d**) C2.

Strain data coming from distributed sensing are expressed in two domains: length and time (load steps). Therefore, they can be conveniently presented within the 3D plots to show both the profile shape over the beam’s length and its development throughout all steps, up to achieving the extreme values in the last step. Such plots are presented in Figure 24.

The last task during the global analysis of strain profiles over length was to compare the locations of crack-induced strain peaks with external observations by metric tape. For all the DFOS tools, except cable C1, very good compliance was obtained. It can be concluded that with the use of appropriate tools, it is possible to locate cracks with accuracy no worse than applied spatial resolution. In the study in question, it was 10 mm.

### 4.2. Crack Width and Shape Coefficient—Definition

The statistical analysis of strain profiles over the entire structural length, presented in the previous section, took into account multiple cracks located close to each other. Although it provided important insights into the measurement quality of applied DFOS tools, it did not consider the analysis of individual cracks.

During this type of comparative analysis, certain evaluation criteria should be defined. The basic parameter is a crack width. In the literature, there are a few procedures describing how to calculate it based on measured distributed strains. The most straightforward approach will be used hereafter, which involves summing the strains *ε* over the effective length *l_eff_*, according to Equation (2).

(2) $$w_{c}=\overset{x_{2}}{\underset{x_{1}}{\sum }}\varepsilon_{i}\cdot sr,\;x_{2}-x_{1}=l_{eff}$$

**Figure 25:**
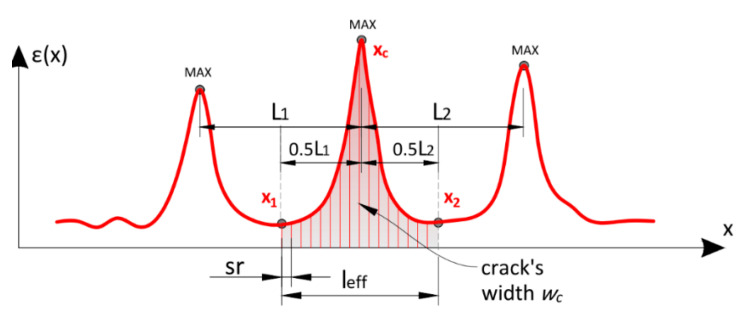
Scheme of calculation of the crack width using conservative approach.

It is a conservative treatment that assumes concrete works in the stabilised crack state (with all possible cracks developed) and does not transfer any tensile strain. Therefore, concrete is considered to have zero strain between cracks, which does not have to be always true (compare Figure 4b). Thus, calculated crack widths could be slightly overestimated. The remaining question is how to adopt the effective length. The study considered multiple cracks located close enough to each other that their lengths overlap. In such cases, half of the spacing on both sides of the analysed cracks can be defined. The graphical representation of that approach, as well as an explanation of the symbols used in Equation (2), are presented in Figure 25. The effective length is approximately equal to the mean spacing of the multiple cracks. In further calculations, this value was defined at 120 mm.

It is also worth noticing that the sensitivity analysis of the obtained crack widths to different calculation models (algorithms) will be the subject of the upcoming articles.

Crack width is one of the most important parameters in the analysis of the quality of selected DFOS tools; however, it alone may not be sufficient. For example, even before calculating the width of the crack, its location must be known. Layered cables experiencing strong internal slippage of the measuring fibre are particularly exposed to the risk of not identifying the presence of a crack. Visual assessment (compare Figure 22b) leaves no doubt, but it was necessary to propose certain mathematical parameters that could be used to evaluate the shape of the strain profile in the context of possible crack detection.

First of all, the analysis is now limited only to the effective length of the crack in question. Secondly, the parameter must express the sharpness or smoothness of the strain profile. As before, therefore, statistical parameters can be used. Standard deviation is an absolute value and does not provide knowledge about the mean. On the other hand, the variation coefficient divides the deviation by the mean value and can be expressed as a percentage (as a relative value). Thus, it was decided to define the parameter called *crack shape coefficient* (*CSC*) as the strain variation over the effective length. From a mathematical point of view, Equation (1) can be used, where the length of the entire beam, *L,* has been replaced by the effective length *l_eff_*:

(3) $$CSC=\frac{stdv_{l_{eff}}}{\varepsilon_{m,l_{eff}}}=\frac{1}{\varepsilon_{m,L}}\cdot \sqrt{\frac{\sum_{i=1}^{K=l_{eff}/sr}\;{\left(\varepsilon_{i}-\varepsilon_{m,l_{eff}}\right)}^{2}}{K-1}}$$

**Figure 26:**
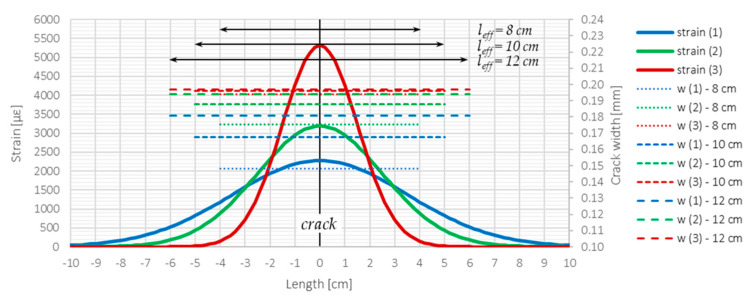
Graphical presentation of different strain profiles and corresponding parameters related to crack analysis (compared to Table 4).

The crack shape coefficient can be calculated separately for each of the selected DFOS tools to compare their quality in crack detection and width estimation. However, before calculating actual measurement data, consider a theoretical example to understand the physical interpretation of the proposed coefficient. Figure 26 presents three examples of crack-induced strain profiles: (1) smoothest, (2) medium, and (3) sharpest.

On the right vertical axis, the crack widths calculated over three effective lengths (8, 10, and 12 cm) are presented. It can be seen that despite significant differences in the shape of the strain profiles, the analysed cases give a range of widths from approximately 0.15 mm to 0.20 mm. The maximum difference is less than 0.05 mm—such an error, from an engineering point of view, could be treated as reasonable. Even assuming a straight horizontal line as a strain profile, it would be possible to calculate the width. That is why it cannot be used as the only parameter to compare the quality of particular DFOS tools.

Table 4 summarises the statistical parameters, including mean value, standard deviation, crack width, and crack shape coefficient, calculated for three example profiles and three assumed effective lengths. On the other hand, Table 5 gives the absolute and relative changes in crack widths and crack shape coefficients while increasing the effective length from 8 cm to 10 and 12 cm. Based on the analysis of provided data, the following insights can be made regarding the analysis of the individual crack and the CSC parameter:

1. The sharper the strain profile, the lower the risk of not detecting the crack.
2. The longer the effective length, the wider the calculated crack widths, but this relation is not linear.
3. The sharper the strain profile, the higher the value of CSC and, thus, the better the DFOS tool for crack detection and width estimation.
4. The higher the value of CSC, the lower the influence of change in effective length on calculated crack width and, thus, the lower the uncertainties of the assumed model.
5. The higher the value of CSC, the lower the influence of change in effective length on CSC value and, thus, the lower the uncertainties of the assumed model.
6. The value of CSC is much more sensitive to changes in the sharpness of the strain profile than the value of crack width.
7. CSC value, together with crack width, could be used as diagnostic parameters to compare the quality of different DFOS tools in the analysis of cracks in concrete based on the measured strain profiles.

### 4.3. Tension Zone—Analysis of Individual Cracks

In this section, the above theoretical information on the calculation of crack widths and corresponding crack shape coefficients are applied based on actual measurement data. Indications of all installed DFOS tools will be compared while analysing the individual cracks—five of them were selected according to Figure 21.

Due to the large number of measurement steps during the test, it was decided to present detailed results from one of the last loading steps, no. 53, at a force equal to 200 kN. However, example data presented throughout the entire research are discussed hereafter.

Table 6 shows calculated widths based on the measured strain profiles. Reference readings on a side surface (REF) were also provided, but they cannot be treated as target values for direct comparison. The course of the crack along the beam’s width is, indeed, randomly variable. However, reference data give a general overview of the external observations performed through conventional techniques. In Table 6, mean values of crack widths and values normalised to unity are also shown. It can be seen that maximum widths were obtained for sensor M2, while the smallest, less by 14%, for cable C1. However, the differences between the reference readings and those obtained from DFOS tools did not exceed 0.04 mm. Thus, it should be stated that, in terms of crack widths calculations, all of the investigated tools provided reliable results.

However, the calculation of crack widths for cable C1 was possible only provided that the location of the particular crack was known, as indicated by the other three tools. Without other tools or external observations under laboratory conditions, such analysis could not be properly implemented. Mathematical proof for that statement is the value of CSC equal to 0.04, which means negligible variability of strains over effective length. The highest value, on the other hand, was obtained for flexible monolithic sensors M2, 0.82, meaning strong strain variation. For cable C2 and sensor M1, intermediate values were calculated, 0.52 and 0.35, respectively.

**Figure 27:**
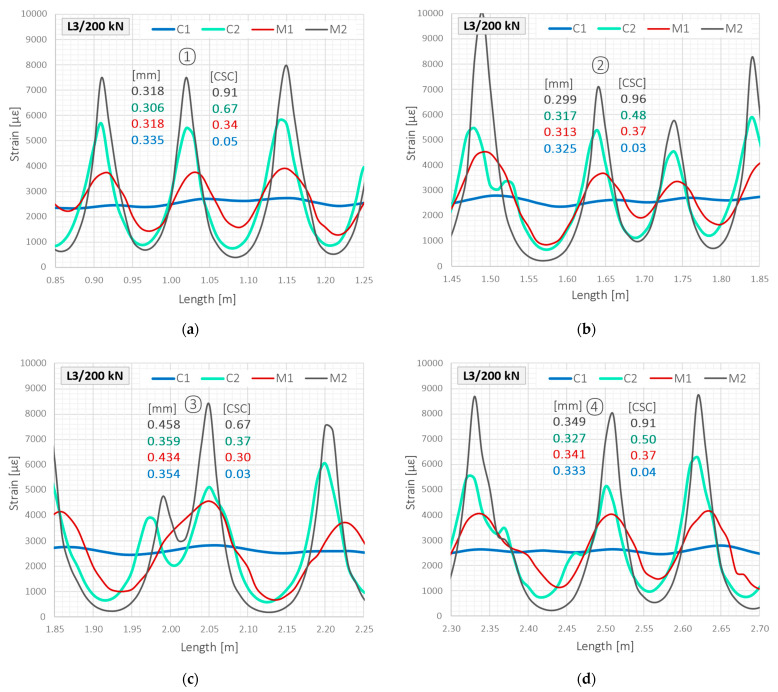
Beam L3/tension zone: close-up to the strain peaks in the load step no. 53 and force of 200 kN for: (**a**) crack no. ①; (**b**) crack no. ②; (**c**) crack no. ③; (**d**) crack no. ④.

Figure 27 shows the data from Table 6 above the close-ups of the strain profiles around the first four analysed cracks. Despite the slight differences in the calculated crack widths, the shapes of the graphs and the values of CSCs unequivocally exclude the possibility of using cable C1 for crack detection in concrete structures. The steel layer around the optical fibre, aimed to increase the mechanical strength of the entire cable, causes uncontrolled internal slippage and strain transfer disturbances, averaging strains along the length. The other tools, although variously sensitive to the presence of a crack, allow for correct interpretation of the structural performance.

**Figure 28:**
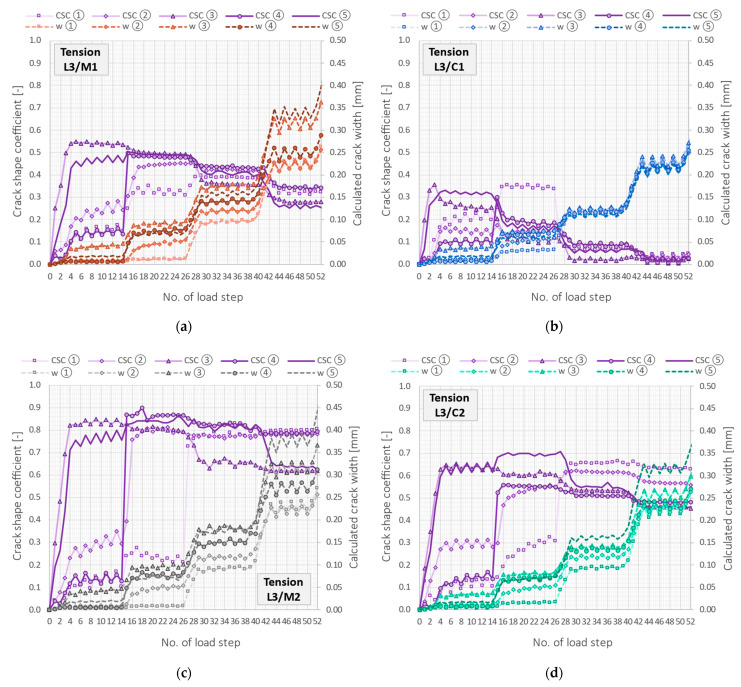
Beam L3/tension zone: crack shape coefficients (purple lines) and widths of five selected cracks calculated based on the following DFOS tools: (**a**) M1; (**b**) C1; (**c**) M2; (**d**) C2.

The above considerations were focused only on the selected load step at force 200 kN. It was just before the structural failure, so this case can be treated as one of the extreme ones. Long-term monitoring of concrete structures cannot be excluded, especially since the measured strains were less than 10,000 µε, which is within the acceptable ranges declared in Table 2 for all the DFOS tools. However, valuable insights can also be made while considering the changes in CSC values throughout the subsequent load step during the entire research. These are presented in Figure 28 with purple lines, together with corresponding crack widths. The best performance was again demonstrated by the monolithic sensor M2 (Figure 28c). From the very first steps corresponding to the formation of the first cracks, the values of CSC were between 0.7 and 0.9. While increasing the load and crack widths, a slight decrease in value was observed because of the elongation of debonding length between the sensor and concrete. However, CSCs were never less than 0.6, while most of them were kept at 0.8.

The values of CSC for cable C2 (Figure 28d) were between 0.4 and 0.7, while for sensor M1 (Figure 28a), they were between 0.3 and 0.6, with a tendency to decrease in value when the crack’s width increases. The performance of these two tools was similar, but cable C2 was more sensitive to crack detection due to its low axial stiffness and good bonding of all internal layers to each other. The monolithic sensor M2, on the other hand, has very high stiffness (both diameter and elastic modulus) and was a local reinforcement for the concrete.

The cable C1 behaved quite differently (Figure 28b). In the initial load steps, CSC values usually did not exceed 0.3, while in the following steps, they systematically decreased, finally reaching values below 0.05. In practice, this means almost complete separation of the fibre from the surrounding layers and, thus, the monitored concrete. By considering the above insights, it could be concluded that despite all of the selected DFOS tools being theoretically designed for the same purpose, which is strain sensing, their performance is significantly different. That is why they cannot be used interchangeably.

First of all, the reduction in axial stiffness of the DFOS tools and slippage phenomena is favourable in terms of sensitivity to crack detection. That is why flexible monolithic sensor M2 could be treated as a universal tool for that purpose. All analysed parameters indicate its best performance throughout all the load steps. Furthermore, it is resistant to local pressures and waving, so no problems were noted during the installation.

The second tool providing valuable strain profiles for crack analysis was cable C2. The good bonding between the particular layers inside the cable allowed for avoiding the extensive internal slippage in the range of 10,000 µε. However, crack-induced strain values in concrete structures can be even higher [63,77]. In work [12], where example results from an S-type beam were presented, the slippage in cable C2 was also reported after exceeding 1% with wider cracks. During the installation, noticeable waving was observed, which can cause additional uncertainties related to the deviations of the cable from the designed path. Moreover, local pressures by cable ties caused disturbances during the early-age concrete measurements [12]. Thus, it can be summarised that cable C2 is a good tool for measuring cracks in concrete, but only within the limited (predicted) range of strains up to 1% and with special care during installation. In practice, this comes down to applications in laboratory conditions.

Monolithic sensor M1 also allowed for clear identification of cracks and correct assessment of the structural behaviour. It was less sensitive to the presence of cracks because of its high axial stiffness and strength, causing the local strengthening of the concrete. For larger and stiffer structural members or entire structures, this effect should be less important. This sensor is the most robust of all the investigated tools, and during installation, it kept a straight line even when increasing the spacing of cable ties used to stabilise it to the stirrups. On this basis, it can be used for applications within real engineering structures and avoided in small-scale laboratory components.

Finally, layered cable C1 with steel strengthening insert was proved to be unsuitable for measuring cracks in concrete, even though the measured strains did not exceed the declared maximum level. Intense slippage of the fibre between the intermediate layers inside the cable did not allow for a correct analysis of the structural behaviour and unambiguous detection of cracks. Such misinterpretation is unacceptable in terms of structural health monitoring goals.

## 5. Field Proofs

**Figure 29:**
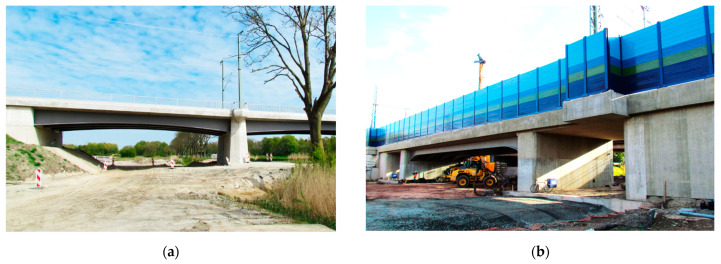
Two German bridges equipped with flexible monolithic DFOS sensors: (**a**) near the city of Sande; (**b**) near the Frankfurt am Main.

This section is focused on two field proofs, where flexible monolithic sensors M1 were embedded in two steel-concrete railway bridges in Germany. The first one is located in the north, near the city of Sande (Lower Saxony) (Figure 29a). The second is in the north of Frankfurt am Main (Figure 29b).

**Figure 30:**
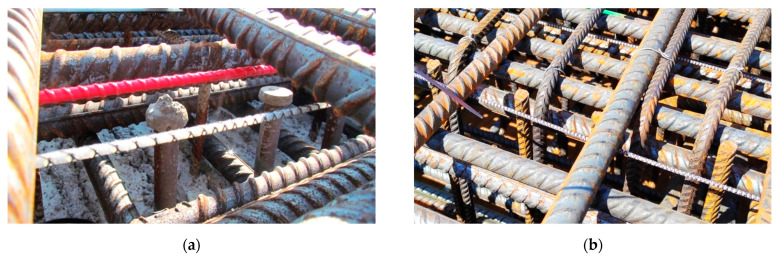
Installation of flexible monolithic DFOS sensors by tying them to the existing reinforcement in two German bridges: (**a**) near the city of Sande; (**b**) near the Frankfurt am Main.

The monolithic sensors were delivered on-site in coils cut at the desired lengths. They were installed inside the slabs before concreting by tying them to the existing reinforcement by cable ties. Example photos from the installation stage are presented in Figure 30a,b, respectively, for these two bridges. Despite challenging construction site conditions, all sensors survived the installation and concreting process, including the process of compacting the concrete by vibration.

**Figure 31:**
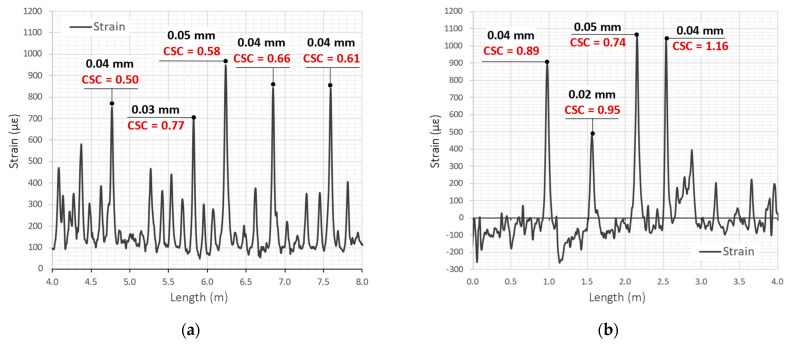
Example analysis of crack widths and crack shape coefficients based on measured strain profiles in the bridges in question: (**a**) near the city of Sande; (**b**) near the Frankfurt am Main.

The DFOS measurements began shortly after concreting so that it was possible to analyse the early-age concrete and the (micro)cracks caused by thermal-shrinkage strains. Currently, the measurements are performed periodically according to the planned schedule. Example strains (compensated due to temperature) along selected 4 m long sections are presented in Figure 31a,b for the two bridges, respectively. The proposed method of crack description, based on its width and CSC, was applied for the first time in real DFOS-based projects. In both new bridges, the calculated crack widths did not exceed 0.05 mm, which is much below the acceptable limits. Cracking is a natural behaviour of reinforced concrete structures, and usually, it is not possible to avoid it. However, the role of the civil engineer is to control the crack widths’ development over time in reference to the defined thresholds. The proposed DFOS-based system allowed for achieving this goal, providing high-quality data. The values of crack shape coefficients are between 0.5 and 1.2, proving the perfect bonding of the monolithic sensors and the concrete, lack of internal slippages, and ability of the system for precise detection of all localised events and discontinuities, even of a very small size.

The example cracks analysis presented in Figure 31 relates only to microcracks formed in early-age concrete (during hydration). By taking into account mechanical loads and possible future degradation of any concrete structure, the measured strain values can be much higher in practice. The DFOS system should be ready to analyse structural behaviour correctly in a near-to-failure state to provide early warning. That is why high sensitivity to crack detection, characteristic of flexible monolithic sensors, is significant and sometimes even safety-critical.

## 6. Conclusions

Cracking in concrete structures is a normal process that starts from the very first days after concreting. The civil engineer’s goal is to monitor and control the crack locations and widths. The DFOS techniques are increasingly used to analyse the behaviour of cracked concrete structures. The ability to perform distributed strain measurements allows for meeting one of the essential objectives for SHM: direct cracks detection and estimation of their widths. This is especially important where visual inspection and assessment are impossible, such as inside the concrete elements or within the components hidden by others (e.g., bridge concrete slabs are usually covered with asphalt layers).

**Figure 32:**
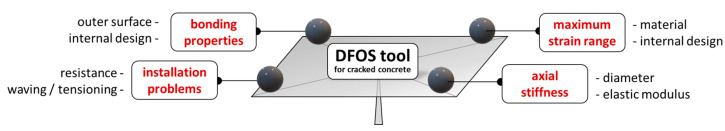
Issues that should be addressed while selecting the appropriate DFOS tool for monitoring the cracked concrete structures.

In order to utilise the unique features of DFOS technology, a set of requirements must be understood and implemented to obtain high-quality data. Some of them are related to the DFOS tools, which, despite being designed for the same purpose (strain sensing), are quite different in their performance. The choice of a particular cable or sensor should always be conscious and preceded by a detailed analysis of the advantages and possible limitations. The presented research shows that finding the optimal solution is about finding the balance between the low axial stiffness, maximum strain range, good bonding properties, and installation issues (Figure 32).

The lower the axial stiffness (cross-section area and elastic modulus), the better the sensitivity of the DFOS tool for crack detection. Moreover, the better the bonding between the measuring optical fibre and the concrete, the better the sensitivity of the DFOS tool. Bonding properties depend both on the outer surface (smooth or rough) and internal design (layered or monolithic). Additional layers inside the cable create the risk of slippage and averaging (smoothing) the strain profile over length. On the other hand, the better the bonding, the higher the crack-induced strain peaks and, thus, the higher the risk of fibre breakage. That is why the high strain range is a very important feature of the DFOS tool. Furthermore, installation issues, such as waving or mechanical resistance, must also be considered.

The extended research indicated the best performance of monolithic and flexible sensors, which could be used as a universal tool for crack detection and analysis, both in the laboratory and field applications. The stiffer monolithic sensors could be used only in real engineering structures, as they could be a local reinforcement for small-size laboratory specimens. The behaviour of layered cables should always be analysed individually. Those flexible and with good bonding between the particular layers could be successfully used within the declared strain range, usually not exceeding 1% in tension. Due to their low resistance to local pressures and extensive waving, they should be installed under strictly controlled conditions, preferably in the laboratory.

Layered cables reinforced with steel inserts, which aim to increase the strength and resistance of the entire cable, are suspected of intensive internal slippage and, thus, disturbances in the strain transfer mechanism. As a result, structural strains are averaged over the entire length so that it is not possible to detect local crack-induced strain peaks. Their application for monitoring cracked concrete structures is not acceptable, as it creates the risk of misinterpretation of structural behaviour and safety.

The versatility of distributed fibre optic sensing extends to the ability to adopt different tools, data loggers, and installation methods to meet the individual requirements of a given project. The lessons learned about different DFOS tools during the research and field proofs are a part of a worldwide discussion about the best configuration of the system designed for a specific purpose: crack detection and analysis within concrete structures.

## Figures and Tables

**Table 1:** Comparison between the general features of layered cables and monolithic sensors (adopted based on [12]).

Parameter	Layered Cables	Monolithic Sensors
Material	plastic, steel	FRP composite
Cross-section	layered	monolithic
Slippage phenomena ^1)^	possible	no
Strengthening inserts	possible	no ^2)^
External surface ^3)^	smooth (usually)	rough (braided)
Elasticity of modulus	unknown ^4)^	specified
Pre-tensioning required ^5)^	usually	no
Maximum strain range	up to ± 1%	up to ± 4%
Indicating the actual state of the structure	depends on model	yes
Adequate for measuring cyclic changes	depends on model	yes
Event memory	depends on model	no
Adequate spatial resolution ^6)^	0.2 m or more	any ^7)^
Design	custom-designed ^8)^	universal ^9)^
Delivered in coils or sections of any length	yes	yes

^1)^ Refers to the slippage inside the sensor (between the internal layers). ^2)^ FRP is more robust than steel or plastic and does not need to be additionally strengthened. ^3)^ External surface influences the bonding properties between the tool and the structure. ^4)^ Reliable estimation of the elasticity modulus of layered cable as a whole is problematic because of the different material properties of particular layers and unknown adhesion and slippage between them. Producers do not provide this information. ^5)^ In the case of measuring strains in the compression zone. ^6)^ Most datasheets suggest the application with Brillouin scattering. For measuring cracks in concrete, at least cm-order spatial resolution is required. ^7)^ Depending on the applied optical data logger. ^8)^ Geometry, the number of layers and their materials should be custom-designed depending on the predicted strain level and occurring threats within the specific application. ^9)^ Only the axial stiffness should be adjusted depending on application (to be reinforcement or not).

**Table 2:** Comparison between the parameters of selected DFOS tools.

Parameter	L1	L2	M1	M2
Strain resolution	-	-	±1 µε	±1 µε
Maximum strain	10,000 µε (1%)	10,000 µε (1%)	±20,000 µε (±2%)	±40,000 µε (±4%)
Nominal diameter	Ø3.2 mm	Ø2.8 mm	Ø5.0 mm	Ø3.0 mm
Elastic modulus	-	-	50 GPa	3 GPa
Axial stiffness EA	-	-	982 kN	21 kN
Material	plastic, steel	plastic	composite G-FRP	composite PL-FRP
Cross-section	2 pl. layers + steel insert	3 pl. layers	monolithic	monolithic
Outer surface	perforated groves	smooth	unidirectional braid	bi-directional braid
Bending radius	48 mm	42 mm	350 mm	50 mm
Sensor weight	10.5 kg/km	5.9 kg/km	45.0 kg/km	13.0 kg/km
Fibre type	SM 9/125	SM 9/125	SM 9/125	SM 9/125
Attenuation	≤0.5 dB/km	≤0.5 dB/km	≤0.5 dB/km	≤0.5 dB/km
Light scattering	Compatible with data loggers based on Rayleigh, Brillouin, and Raman scattering			
Delivery method	coils or straight sections			
Length	any length made to order			

**Table 3:** Global statistical parameters (mean, maximum, standard deviation, and variation coefficient) for all investigated DFOS tools in selected load steps.

**Load Step**	**Force**	**Mean Strain (µε)**				**Maximum Strain (µε)**			
		**C1**	**C2**	**M1**	**M2**	**C1**	**C2**	**M1**	**M2**
16	60 kN	438	432	433	434	901	1938	1613	2737
29	90 kN	897	886	892	912	1319	3297	2438	4097
41	120 kN	1319	1318	1317	1319	1707	4285	3144	5963
43	160 kN	1876	1874	1884	1884	2233	5630	4088	7997
53	200 kN	2417	2411	2427	2430	2832	6852	5080	10,053
Mean values		1390	1384	1391	1396	1799	4400	3273	6169
Normalised values		1.00	0.99	1.00	1.00	0.29	0.71	0.53	1.00
**Load Step**	**Force**	**Standard Deviation (µε)**				**Variation Coefficient [%]**			
		**C1**	**C2**	**M1**	**M2**	**C1**	**C2**	**M1**	**M2**
16	60 kN	246	449	391	571	56	104	90	132
29	90 kN	285	790	640	1036	32	89	72	114
41	120 kN	292	1042	805	1383	22	79	61	105
43	160 kN	297	1325	964	1839	16	71	51	98
53	200 kN	341	1602	1093	2324	14	66	45	96
Mean values		292	1042	779	1431	28	82	64	109
Normalised values		0.20	0.73	0.54	1.00	0.26	0.75	0.59	1.00

**Table 4:** Statistical parameters of individual cracks calculated based on the three example strain profiles for three different effective lengths (see Figure 26).

Eff. Length	Parameter	Example (1)	Example (2)	Example (3)
8 cm	mean (µε)	1850	2193	2424
	stdv (µε)	353	764	1816
	width (mm)	0.148	0.175	0.194
	CSC (-)	0.19	0.35	0.75
10 cm	mean (µε)	1676	1880	1959
	stdv (µε)	476	935	1880
	width (mm)	0.168	0.188	0.196
	CSC (-)	0.28	0.50	0.96
12 cm	mean (µε)	1506	1615	1639
	stdv (µε)	582	1043	1865
	width (mm)	0.181	0.194	0.197
	CSC (-)	0.39	0.65	1.14

**Table 5:** The changes in crack widths and crack shape coefficients depending on the change in the assumed effective length (see Figure 26).

Parameter	Example	Absolute Increase		Percentage Increase	
		8 → 10 cm	8 → 12 cm	8 → 10 cm	8 → 12 cm
Crack width	(1)	0.020 mm	0.033 mm	13.3%	22.1%
	(2)	0.013 mm	0.018 mm	7.2%	9.5%
	(3)	0.002 mm	0.003 mm	1.1%	1.4%
Crack shape coefficient	(1)	0.09	0.20	48.7%	102.5%
	(2)	0.15	0.30	42.8%	85.4%
	(3)	0.21	0.39	28.1%	51.9%

**Table 6:** Calculated crack widths and crack shape coefficients for all the DFOS tools in load step no. 53 and force of 200 kN.

Crack	Width Change (mm)					Crack Shape Coefficient (-)			
	C1	C2	M1	M2	REF	C1	C2	M1	M2
①	0.335	0.306	0.318	0.312	0.26	0.05	0.67	0.34	0.91
②	0.325	0.317	0.313	0.299	0.30	0.05	0.48	0.37	0.96
③	0.354	0.359	0.434	0.458	0.40	0.03	0.37	0.30	0.67
④	0.333	0.327	0.341	0.349	0.32	0.02	0.50	0.37	0.91
⑤	0.324	0.425	0.493	0.531	0.56	0.04	0.60	0.36	0.68
Mean	0.334	0.347	0.380	0.390	0.368	0.04	0.52	0.35	0.82
Norm.	0.86	0.89	0.97	1.00	-	0.04	0.64	0.42	1.00

## Data Availability

Data are available on request from the authors.
